# The temporal visual oddball effect is not caused by repetition suppression

**DOI:** 10.3758/s13414-023-02730-4

**Published:** 2023-07-06

**Authors:** Blake W. Saurels, Kielan Yarrow, Ottmar V. Lipp, Derek H. Arnold

**Affiliations:** 1 School of Psychology, The University of Queensland, St Lucia, Australia; 2 Department of Psychology, City, University of London, London, UK; 3 School of Psychology and Counselling, Queensland University of Technology, Brisbane, Australia

**Keywords:** Oddball, Perceived duration, Repetition suppression, Prediction, Anticipation

## Abstract

The oddball paradigm is commonly used to investigate human time perception. Trains of identical repeated events (‘standards’) are presented, only to be interrupted by a different ‘oddball’ that seems to have a relatively protracted duration. One theoretical account has been that this effect is driven by repetition suppression for repeated standards. The idea is that repeated events seem shorter as they incur a progressively reduced neural response, which is supported by the finding that oddball perceived duration increases linearly with the number of preceding repeated standards. However, typical oddball paradigms confound the probability of oddball presentations with variable numbers of standard repetitions on each trial, allowing people to increasingly anticipate an oddball presentation as more standards are presented. We eliminated this by making participants aware of what fixed number of standards they would encounter before a final test input and tested different numbers of standards in separate experimental sessions. The final event of sequences, the test event, was equally likely to be an oddball or another repeat. We found a positive linear relationship between the number of preceding repeated standards and the perceived duration of oddball test events. However, we also found this for repeat tests events, which speaks against the repetition suppression account of the temporal oddball effect.

**Supplementary Information:**

The online version contains supplementary material available at 10.3758/s13414-023-02730-4.

## Introduction

The oddball paradigm involves presenting a sequence of identical repeated events that are broken by a surprising ‘oddball’. This paradigm has been used to examine the effect of repetition and surprise on perceived duration. It has been shown that oddballs seem longer than repeated ‘standards’—the temporal oddball effect (Pariyadath & Eagleman, 2007; Saurels et al., 2019; Tse et al., 2004). There are competing accounts regarding the cause of this likely multifaceted effect (see Ulrich & Bausenhart, 2019, and Killeen & Grondin, 2022, for recent overviews of timing and time perception research).

One suggestion is that the effect is tied to repetition suppression (Matthews, 2015; Pariyadath & Eagleman, 2007, 2012)—a reduction in neuronal responsivity to repeated stimuli (Buckner et al., 1998; Grill-Spector et al., 1999). Pariyadath and Eagleman (2012) examined this using an oddball experiment, where the oddball was presented after a variable number of standard repeats (1–5). They found a positive linear relationship between numbers of standard events preceding an oddball and the perceived relative duration of oddballs (see Supplemental Figure 1). They suggested this effect might be a consequence of repeated events seeming to have a progressively shorter duration because of repetition suppression—the tendency of the human brain to be less responsive to repeated inputs (Buckner et al., 1998; Grill-Spector et al., 1999).

Pariyadath and Eagleman’s (2012) findings could also be explained by differences in attentional allocation driven by anticipation. In this type of protocol, an oddball will eventually be presented in each trial sequence, so as participants see more repeats, they might appreciate that the likelihood of seeing an oddball on the next presentation has increased. Formally, we can say that while the distribution of oddballs across trial sequence positions is uniform, the hazard function is not. Participants could therefore (consciously or unconsciously) anticipate a rising need to deploy attention to an oddball event—in order to accurately gauge its duration.

**Fig. 1:**
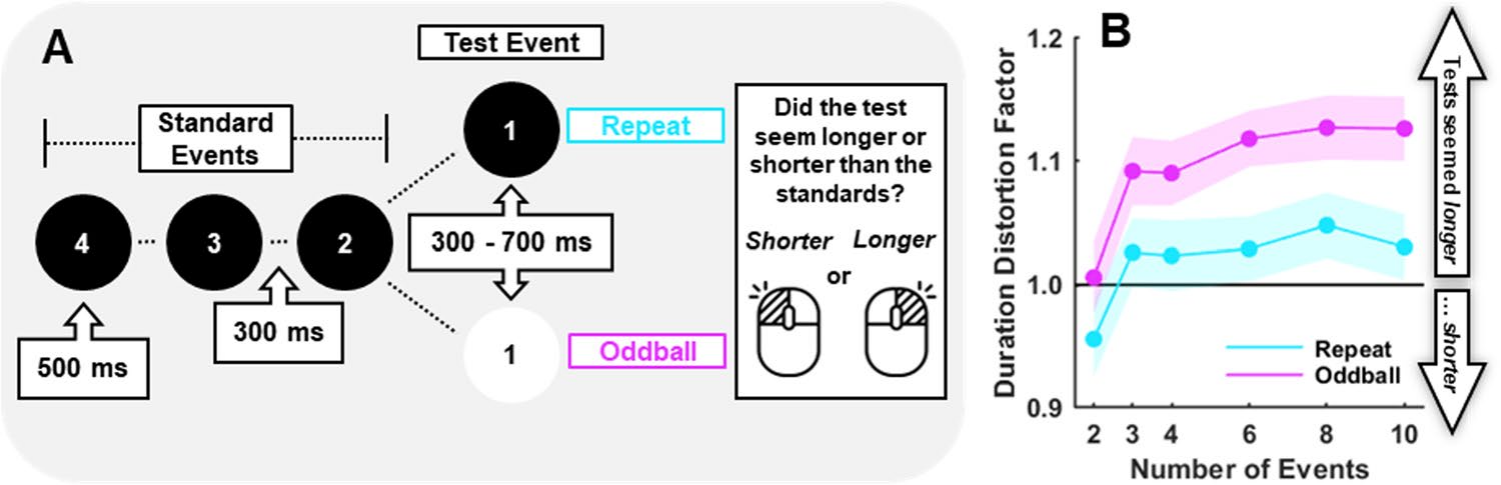
**A** Experimental paradigm. Illustrates the trial sequence from 4-event session trial. **B** Mean DDF for each trial type as a function of the number of events. A DDF greater than 1 indicates that test events seemed longer than standard events, and vice versa. Shaded error bars depict ±1 standard error amongst participants. (Colour figure online)

To disambiguate the findings of Pariyadath and Eagleman (2012), we created an experiment to assess the impact of seeing different numbers of repeated events before an oddball, while controlling for potential attentional differences as people anticipate an oddball. We had participants perform six sessions of an oddball task, where they had to compare the final event in each sequence (the ‘test’ event) to the duration of preceding repeated standards. The number of events they would see on each trial was constant within each experimental session, but was varied across sessions, so participants always knew when they would see the test event on each trial. We also numbered all events shown onscreen as a countdown to the test presentation (see Fig. 1A; this is similar to an approach used by Birngruber et al., 2015, where standard event numbers were known by the participant and signalled by a simultaneously flashed circle that shifted in a clockwise fashion). Test events were equally likely to be an oddball, or another identical repeat. This allowed us to also test if oddball test events were perceived differently than repeated events, even when they could be equally anticipated (this method is used in Birngruber et al., 2015, and in Saurels et al., 2019).

The repetition suppression account suggests a progressive decrease in the perceived duration of repeated standards, with a relative increase for oddball test events. This predicts that as people see more standards, the relative perceived duration of oddballs should progressively increase, and the relative perceived duration of repeat tests should progressively decrease. The results of Pariyadath and Eagleman (2012) suggest these changes should be linear.

## Methods

### Participants

Eighteen volunteer participants were recruited for testing via a research participation scheme at the University of Queensland (in exchange for course credit), as well as two naïve lab members—for a total of 20 participants (three male). For the conceptually central effect of varying the number of events, one of the closest studies to ours had a *n* of 11 (Pariyadath & Eagleman, 2012) but provided insufficient information to determine effect size—thus, an *n* of 20 seemed sufficient to provide enough power. For the second effect of interest here (oddball vs. repeat tests) Birngruber et al. (2015) Experiment 3 is relevant and based on the effect size reported there our *n* of 20 yields 86% power (two-tailed paired *t* test, alpha = .05). All participants reported having normal or corrected-to-normal visual acuity. Ages ranged from 18 to 26 (*M* ~ 19, *SD* ~ 1.8). Participants were informed that they could withdraw from the study at any time without penalty. The experiment was approved by the University of Queensland ethics committee and adheres to the Australian Code for the Responsible Conduct of Research.

### Stimuli and apparatus

Stimuli were black (CIE: 0.272, 0.376, 5.016) or white (CIE: 0.285, 0.322, 138.05) circles presented against a grey background (CIE: 0.278, 0.333, 37.772). Circles had a diameter subtending ~10 degrees of visual angle. Stimuli were presented on a calibrated 20-in. CRT SONY Multiscan G420 monitor, driven by a Psychtoolbox-3 (Kleiner et al., 2007) and custom MATLAB R2020b software (The MathWorks, Natick, MA). The monitor had a resolution of 1,024 × 768 pixels and a refresh rate of 60 Hz. Participants viewed stimuli from 57 cm directly in front of the monitor, with their chin placed on a chin rest.

### Design and procedure

The experiment was split into six blocks that differed in the number of sequential circle presentations per trial (2, 3, 4, 6, 8, or 10). An example of a four-event trial sequence is depicted in Fig. 1A. We refer to the last event of each sequence as the ‘test’ event, and preceding events as ‘standards’.

Event timings were matched to Pariyadath and Eagleman (2012). All standard events persisted for 500 ms and tests varied between 300 and 700 ms, in 50-ms intervals (nine possible test durations, equiprobable and presented in a random order). Presentations were separated by a 300-ms interstimulus interval (ISI). Events were numbered in the centre, with numbers doubling as a fixation point. This provided a count-down for participants, so they always knew when the test event, which was key to the experimental task, would be presented. Participants were encouraged to attend to all events, so that they could get a good impression of the duration of standards.

On 50% of trials the test event was a different colour (an ‘oddball’) relative to preceding standards. Event colours (black or white) were counterbalanced within each block of trials and were presented in a randomized order.

At the end of each trial, participants were asked if they thought the test had seemed to last longer (right click) or shorter (left click) than standards. There were a total of 180 trials in each block (10 for each of the nine test durations for each trial type: repeat and oddball). In total, participants completed 1,080 individual trials, across six blocks of trials completed in a randomized order.

Participants received written and verbal instructions. They completed six trials at the start of the first block, which were set to the easiest difficulty (either 300-ms or 700-ms test events) in order to verify that they had understood task instructions and to check if they could discern a 200-ms duration difference (all participants could). These practice trials had feedback, whereas nonpractice trials did not. Participants were told that there would always be a difference in physical duration between test and standard event durations, but that on some trials it would be very hard to detect. They were also told that there would not necessarily be an equal number of trials where the test event would be shorter or longer than standards. The precise wording for the test used in the written instructions was: *“Your task is to report if the LAST flashed circle in each sequence seemed to be presented for shorter or longer than the ones that came before it”*.

The experiment was therefore a 6 (number of events) × 2 (test event type) repeated-measures design, with the dependent construct being the apparent duration of the test event relative to preceding standards (see Results section for details on how this was quantified).

### Analyses

We calculated the proportion of trials on which participants had reported that the test event seemed longer than standards, for each test duration within each block, separately for repeat and oddball tests. We fit cumulative gaussian functions using psignifit (Schütt et al., 2016) to these data, and from these calculated the point of subjective equality (PSE) between standard and test durations (i.e., the point at which participants reported that test events had seemed longer than standards on 50% of trials). We then calculated ‘Duration Distortion Factors’, or DDFs, by taking the ratio of the standard duration (500 ms) to the PSE for test events (as per Pariyadath & Eagleman, 2007, 2012; e.g., with a PSE of 450 ms, you would get a DDF of ~1.11, indicating a duration distortion of +11% for test events). Deviance values were calculated for these fits and are presented alongside plots of the fits and data in Supplemental Figure 2. For subsequent inferences we used two-tailed frequentist alpha of 0.05 and/or Bayesian evidence strength categories consistent with those of Jeffreys (1961/1998). The latter analyses were conducted using a MATLAB toolbox from Krekelberg (2021).

### Transparency and openness statement

All data and code can be found at UQ eSpace.

## Results

### Perceived duration

We examined the relationship between DDFs and the number of repeated events preceding oddball and repeat tests (see Fig. 1B) using a linear mixed model (the same approach as Pariyadath & Eagleman, 2012, except they could only examine this relationship for oddball tests). The model used trial type and the number of events as predictors, as well as their interaction (with separate intercepts and slopes for each participant, i.e., fixed and random effects for all predictors). We found a positive linear relationship between the number of events and DDFs, β_Number of Events_ = 0.009, *t*(236) = 4.36, *p* < .001. We also found that oddball tests (*M* = 1.09, *SD* = 0.09) seemed longer than repeats tests (*M* = 1.02, *SD* = 0.11), β_Trial Type_ = 0.037, *t*(236) = 6.45, *p* < .001. However, we did not find an interaction between the number of events and the trial type, β_Number of Events × Trial Type_ = −0.002, *t*(236) = 1.47, *p* = .142. So, the more repeated standards, the longer the test event seemed—and while oddball tests generally seemed longer than repeat tests, the pattern of change with event number was not significantly different for test types.

Inspection of Fig. 1B suggests that the two-event condition might be distinct from other event number conditions. So, we repeated the above analysis but excluded data from two-event sequences. This analysis produced the same pattern of results; we found a positive linear relationship between the number of events and DDFs, β_Number of Events_ = 0.004, *t*(196) = 2.36, *p* = .019, that oddball tests seemed longer than repeats tests, β_Trial Type_ = 0.04, *t*(196) = 6.37, *p* < .001, and no interaction between the number of events and the trial type, β_Number of Events × Trial Type_ = −0.002, *t*(196) = 1.23, *p* = .221.

As an additional point of clarification, we verified that the difference between repeat and oddball tests was present even in the two-event condition, pairwise comparison statistics: *t*(1,19) = 2.66, *p* = .02, with moderate evidence for the alternative hypothesis, that there would be a difference, *BF*_10_ = 3.55.

We were motivated to examine if perceived duration was exaggerated relative to the objective duration of standards, separately for each combination of Trial Type × Number of Events (as a follow-up to Birngruber et al., 2015). So, we conducted an exhaustive set of Bayesian one-sample *t* tests. These revealed that DDFs for repeat tests were anecdotally no different from 1, regardless of the number of standards (anecdotal to moderate evidence for the null hypothesis, that there would be no difference). However, for oddball tests, DDFs were always greater than 1 (strong to decisive evidence for the alternative hypothesis, that there would be a difference), except for the two-event condition, where they were no different from 1 (moderate evidence for the null hypothesis).

### Duration difference limens

We also checked participants’ ability to discriminate between test durations (see Supplemental Figure 3). To do this, we calculated difference limens (DLs; half the difference between the test duration where 75% of tests were reported as longer and the test duration where 25% of tests were reported as longer, in ms; [(0.75 level − 0.25 level) / 2]). We then used these as the dependent variable within a linear mixed model with the same predictors as the one used for perceived duration. We found a negative linear relationship between the number of events and DLs, β_Number of Events_ = −4.15, *t*(236) = 3.51, *p* < .001. However, we did not find an effect of trial type, β_Trial Type_ = 4.56, *t*(236) = 1.78, *p* = .076, or an interaction, β_Number of Events × Trial Type_ = 1.22, *t*(236) = 1.31, *p* = .192. So, participants were better able to discriminate test durations after seeing more repeated standards, but this effect was no different for repeat and oddball tests.

## Discussion

We found that oddball tests seemed to last longer than repeat tests, and that oddball tests, but not repeat tests, seemed to last longer than repeated standards. We also found that the apparent duration of repeat and oddball tests relative to standards scaled with the number of preceding standards. Crucially, contrary to the predictions of the repetition suppression account of the temporal oddball effect (Pariyadath & Eagleman, 2007, 2012), there was no evidence of a tendency for repeat test events to have an increasingly shortened apparent duration with increasing numbers of repeated standards.

These results are consistent with an impact of repetition, but not repetition suppression as outlined by Pariyadath and Eagleman (2007, 2012), in producing the temporal oddball effect. The repetition suppression account suggests that as people see more repeated standards, their impression of the duration of a further repeat should decrease, in line with a decreased neural response to repeated visual events (Pariyadath & Eagleman, 2007, 2012; Saurels et al., 2019). Encountering an oddball test should break this trend of decreasing neural response, providing a relative increase in perceived duration for oddballs relative to the impression of preceding repeats. However, by the same logic we would expect to see a progressive decrease in perceived duration for repeat tests—which we did not. To be clear, we are not refuting that repetition suppression occurs in oddball tasks, but just refuting that it causes changes in perceived duration in temporal oddball tasks as described above.

So, what is causing the slight increase in apparent duration for test events as people encounter more repetitions? This could be explained by differences in attention—not to the test event itself, but to the preceding repeated standards. Attention has long been known to impact time perception, such that attending to time (prospective judgments) will dilate our experience of it (Block & Gruber, 2014; Brown, 1985; Polti et al., 2018). In our task, test events could *always* be anticipated, so participants were never surprised by the need to attend to the duration of test events—thus, there was no confounding of preparedness for a test event and the number of repeated standards. However, this still allows attention levels to vary for the repeated standards themselves. Perhaps when people knew they would see more repeated standards they paid less attention to the duration of some of them. This would produce a progressive increase in the relative perceived duration of repeat *and* oddball tests, which is what we observed.

In addition to a slight increase in perceived duration with the number of repeated standards, we found that oddballs tests seemed (uniformly) longer than repeat tests. This was despite oddball and repeat tests being equally likely to occur, and participants knowing when the test event would occur. Again, this could be explained by differences in attention—but this time for the test event itself. Perhaps oddballs capture our attention better due to their *oddness*. This aligns with the findings of Tse et al. (2004), who show that oddballs need to be at least ~120-ms long to evoke a duration exaggeration—a temporal profile consistent with how long it takes to ‘capture’ attention (Nakayama & Mackeben, 1989). This capturing of attention by oddballs might be expected to prolong and sharpen temporal experiences. Interestingly, unlike Birngruber et al. (2015—who also made participants aware of when test events would occur, and had repeat and oddball test events, but instead made oddball tests statistically less likely to occur than repeat tests), we did not find that participants were significantly better able to discriminate test durations for oddballs (although we did observe a nonsignificant trend in that direction).

If oddballs draw attention, and attention dilates time, one could then ask what makes oddballs *odd*? Adaptation might seem like an obvious explanation, except that this has been ruled out by Schindel et al. (2011), who showed that a protracted, continuous visual event resulted in Troxler fading (a phenomenon known to result from low-level monocular adaptation; see Clark & Belcher, 1962), but had no impact on the perceived duration of subsequent events.

So, perhaps a more generalized neural predictive processes is at play (Bubic et al., 2010). A violation of a train of repeats could also be considered a violation of a prediction—that simply seeing repetitions makes another repetition seem likely, and therefore makes oddballs *odd*. The predictive coding framework, for instance, stipulates that prediction violating events incur a neural prediction error, and that these events are then subjected to increased processing to update an internal model of the world—to avoid future prediction errors (Rao & Ballard, 1999). This prediction about seeing more repetitions would need to be divorced from the actual statistical likelihood of events though, as repeat and oddball tests were equally likely in our experiment. This account would also need to be reconciled with the finding that events that are actually statistically improbable do not incur an apparent duration distortion (Cai et al., 2015; Saurels et al., 2022).

A final question we wish to address is why the two-event condition is so different? It is known that the initial event of sequences seems to last longer than subsequent repeats (the ‘debut effect’; Pariyadath & Eagleman, 2007; see also Rose & Summers, 1995). In our data, when the initial event was followed by an oddball test, we found that these events were perceptually matched in terms of duration. This could be because, in a sense, both events are odd in this scenario—the first is odd relative to the preceding nothingness, and the second is odd relative to the first. If a repeat test occurs instead, this will seem shorter than if an oddball had been presented. So, the jump in apparent duration between the 2-event and 3-event condition (see Fig. 2B) for both repeat and oddball tests can be attributed to an impact of seeing an actual repeat event (an effect of repetition).

In summary, the results of this experiment support a positive relationship between repetition number and the perceived duration of repeat and oddball test events. This speaks against the repetition suppression account of the temporal oddball effect whereby repeated inputs incur a progressive reduction in perceived duration.

### Supplementary Information

ESM 1 (DOCX 668 kb)
