# Supplementary material for: The temporal visual oddball effect is not caused by repetition suppression
Source: Atten Percept Psychophys. 2023 Jul 6;85(6):1755–60. doi: 10.3758/s13414-023-02730-4 (PMC10545560; doi:10.3758/s13414-023-02730-4)
Supplement: Supplementary file 1 — (DOCX 668 kb) [file 13414_2023_2730_MOESM1_ESM.docx]

**SUPPLEMENTAL**


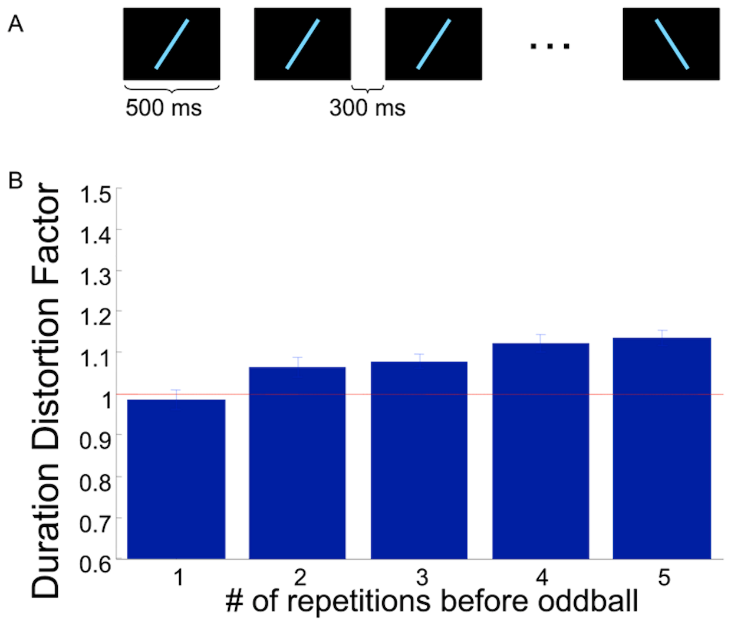


**Supplemental Figure 1.** A copy of Figure 1 from Pariyadath and Eagleman (2012). Their description: “The size of the oddball effect depends on the number of repetitions of standard stimulus. (A) Cartoon depicting the experimental design. Participants viewed a stream of repeated lines with an oddball line that appeared anywhere from the 2nd to the 6th position. (B) The number of repetitions of the standard stimulus modulates the size of the temporal oddball effect.”


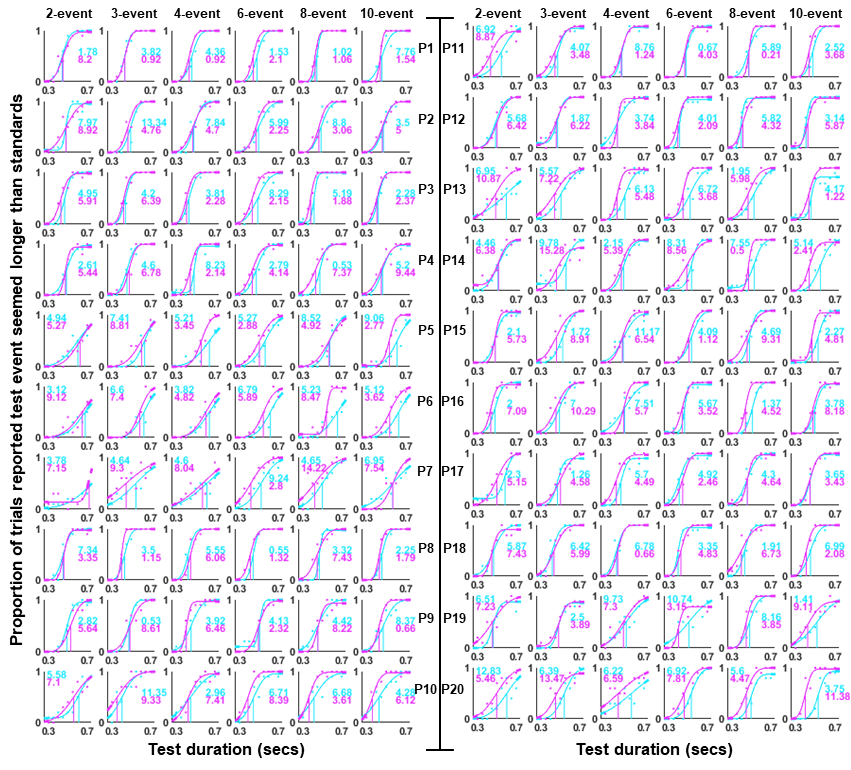


**Supplemental Figure 2.** Coloured dots depict the proportion of trials that participants reported the test duration seemed longer than the standards for each test duration. This is shown separately for repeat (cyan) and oddball (magenta) trial types, for each event number condition (columns), for each participant (rows). Coloured lines depict cumulative gaussian functions fit to these data. Inset coloured numbers depict the deviance values for each function fit, which can be compared against a critical value of 11.07 (alpha = .05) from a chi-square distribution with 5 degrees of freedom. There were 8 fits that exceeded this value (8 out of 240 = 3.33%), which is comparable with the expected 5% rate if the fitted model is the true generative model.


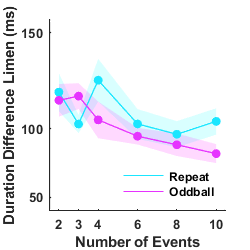


**Supplemental Figure 3.** Test duration discrimination performance (difference limen in ms) as a function of the number of events. A smaller difference limen indicates smaller physical test durations could be distinguished. Shaded error bars depict ±1 standard error amongst participants.
